# 

**Published:** 1895-08

**Authors:** 


### CONTENTS:

### SELECTIONS.

Article I.—Fifty Years in the Laboratory; or The Evolution of the Dental Laboratory as I have seen it. By L. P. Haskell. - - - - - - - - 145
" II.—The Successful Treatment of Cleft Palate. By Eugene F. Hoyt, M. D. - - - - - 154
" III.—Dead Teeth. By John G. Rankin, L. D. S. - 157
" IV.—Nuclein. By Dr. E. Bergstretser. - - - - 164
" V.—The Structure of the Maxillary Bones. By Dr. M. H. Cryer. - - - - - - - - - 175
" VI.—A Digest. By Carl Fischer, D. D. S. - - 179
" VII.—The Dental Filling. By Dr. Joseph Head. - - 181

### EDITORIAL, ETC.

The Teeth of our School Children. - - - - - - 182

### MONTHLY SUMMARY.

Filling Pulpless Teeth with Fistulous Opening. - - - - 182

Friction-Massage with the Fingers. - - - - - - 183

Transmission of Cholera by the House Fly. - - - - - 184

The Preventive Treatment of Seasickness. - - - - - 184

Protecting Pulps. - - - - - - - - - 185

The Application of the Rubber Dam. - - - - - - 185

Lining Rubber Plate with Aluminum. - - - - - - 186

Vulcanizing Between Metal. - - - - - - - 186

Amalgam. - - - - - - - - - - 187

Formula for Pulp Devitalization. - - - - - - 187

Tortuous Canals. - - - - - - - - - 188

Thinking with the Finger Tips. - - - - - - - 188

Erosion. - - - - - - - - - - 188

Antisepsis in Dentistry. - - - - - - - - 189

Decay of the Teeth. - - - - - - - - 189

Extracting an Abscessed Tooth. - - - - - - 190

Root Canals. - - - - - - - - - - 191

Digestion. - - - - - - - - - - 191

Arsenic. - - - - - - - - - - 192

